# Glances at the Hospitals

**Published:** 1902-01-11

**Authors:** 


### GLANCES AT THE HOSPITALS.

### VICTORIA HOSPITAL, BURNLEY.

The income was £3,445, and the expenditure £3,204. The average cost of each bed occupied was £48 5s. The endowment fund has been increased by £4,769, representing the residuary estate of Miss Emma Rothwell. The total number of in-patients was 1,184, the daily average number of beds occupied was 65, and the total number of deaths was 64. A new operation theatre is being built. The medical and surgical tables convey information as to the results of treatment, but in some cases a column for "remarks" would be desirable. For instance, we notice that three cases of aortic aneurism were treated in the medical wards, and that they were all "cured or relieved." What treatment was pursued? Of 15 cases of phthisis we should like to ask the same question. It is evident that much care and time have been expended in the compilation of these tables, and it seems a pity that a little more information could not be given to us. The operation table does give an extra column for particulars. There were 18 amputations and one death, but the latter was owing to internal injuries, the result of the accident.

### KIDDERMINSTER INFIRMARY.

The income was £2,687 and the expenditure was £2,863, showing a deficit of £176. The committee account for this by saying that not only has there been a higher number of patients to deal with, but higher prices have had to be paid for articles consumed in the hospital. Owing to the changes in the system of medical examinations, we are informed, and to the war, no applications were received for the post of house surgeon when a resignation took place, and the institution was for some time without a resident medical officer. At the beginning of the year there were 45 patients in the infirmary, and 482 were admitted during the year, making a total of 527. Of these 322 were discharged cured, 93 relieved, 72 for various reasons, 40 died. Of the latter 8 died within 48 hours of admission. A list of the diseases from which the in-patients suffered is given, and also a list of the operations, but no results are stated. Anæsthetics were administered 275 times, but the kind is not mentioned.

### THE SUNDERLAND INFIRMARY.

The total income was £10,234, which was about £450 less than that of the previous year. It is with pleasure we note that the workpeople's contributions increased by about £170 and reached a total of £6,287. The ordinary expenditure amounted to £9,653, and the extraordinary to £1,339. The total number of in-patients treated during the year was 2,705, and the out-patients 5,907, making a grand total of 8,612, being nearly 1,000 in excess of the previous year. The daily average number of in-patients was 183, the average cost per head was £3 6s. 3d., the cost per bed occupied was £48 15s. 8d., and the average residence in the infirmary was 24½ days. The cost for stimulants for the whole year was only £57 6s. 8d. being an average of 5d. per patient. On page 17 a useful table is given which shows that of 1,527 surgical cases treated during the year 1,149 were cured, 109 relieved, 61 died, 22 were "not improved," 95 were made out-patients, 17 were transferred, and 74 were under treatment at the date of the report. Of 1,178 medical cases, 554 were cured, 324 relieved, 25 died, 64 not improved, 155 were made out-patients, 8 were transferred, and 48 remained in the infirmary. The medical and surgical tables are carefully drawn out and give all necessary information concerning the termination of the cases, but we miss a table showing the nature of the anæsthetics used during the surgical operations; an omission which we hope may be supplied in the report for next year.

### BIRMINGHAM FREE HOSPITAL FOR SICK CHILDREN.

The total income was £4,754 and the total expenditure £5,345, showing a deficit of £590; but the President (Mr. Cadbury) presented £250 to reduce the adverse balance, and we cannot doubt that his example will be followed by others and the whole debt wiped off. The in-patients reached the respectable number of 1,063, the out-patients 13,535, and the casualties 231. The average "detention rate" was 19 days, and the death rate, calculated on the 1,060 patients treated to the termination of the case, was 7·9 per cent. among the in-patients. The Medical Committee point out that the death rate from diphtheria was 41·1 per cent. in 1897, 29·6 per cent. in 1898, 18 per cent. in 1899, and only 15·4 per cent. in 1900. This is full of hope for the future treatment of this disease. It would be a vast improvement if the medical and surgical table were provided with additional columns showing the terminations of the cases. We notice that 39 cases of diphtheria were treated, but we have to refer to another table to learn that there were 7 deaths from this disease, of 15 cases of enteric fever, 1 died; and apparently there was only 1 case of scarlatina which proved fatal. Again, there were 11 cases of tubercular meningitis, and by wandering over the deaths table we find that 8 patients died of that disease, but we are left in doubt about the remaining 3.

### LIVERPOOL ROYAL INFIRMARY.

It is to be regretted that the finances of this large infirmary are not in a very flourishing condition, and the committee state the proportion of subscribers in a city of 700,000 inhabitants falls far short of what it should be. It would seem that a debt of £2,800 exists, and had it not been for the transfer of £2,749 from another fund, that debt would have stood at £5,550. This is certainly not creditable to a rich city like Liverpool, and should it continue must inevitably cripple the usefulness of the hospital. In future reports it would be desirable to know what proportion of the £3,155 received in annual subscriptions, was contributed by the workpeople themselves; what was the cost per head per patient; the cost per bed occupied; and, above all, what is the wage limit above which gratuitous medical advice is refused. We notice that the average duration of the patients' residence was upwards of 29 days. In the Sunderland Infirmary noticed above it was only 24½ days. Were this to be followed in the Liverpool Infirmary it would mean a saving of 14,000 days' maintenance. We notice, also, that over 20,000 out-patients were treated. On January 1st there were 254 patients in the infirmary; 2,870 were admitted during the year, making a total of 3,124. Of these 2,601 were discharged, 264 died, and 259 remained under treatment. The medical and surgical tables are very well arranged. Separate columns show the ages, numbers, cures, relieved, deaths, and not relieved. There is also a useful column of "remarks" given with one of the operation tables.

